# Alex de Waal, *New Pandemics, Old Politics: Two Hundred Years of War on Disease and Its Alternatives*

**DOI:** 10.1093/shm/hkab089

**Published:** 2021-08-14

**Authors:** Ylva Söderfeldt

**Affiliations:** Uppsala University, Sweden

During the coronavirus pandemic, insights from the social history of medicine have been subject to unprecedented public interest. As we live through a pivotal moment in the relationship between disease, politics and society, we recall previous pandemics in attempts at making sense of our experience, at predicting the course of events ahead, and evaluating and guiding the response.

Among a long and growing list of books that set out to find historical lessons in previous pandemics, Alex de Waal, an anthropologist specialised in human rights and humanitarian crises, stands out with his focus on a political narrative we have grown used to that of ‘war on disease’. This is the script that casts healthcare workers as ‘the front line’ and viruses as ‘invisible enemies’. The war script is reassuring and powerful, as it allows the public to visualise an end to the crisis through defeat of the pathogen and like with proper warfare, it provides a justification for foregoing human rights and civil liberties. It is this narrative which de Waals sees as the main impediment on not only just but also on effective pandemic response.

The argument is laid out through a global history of pandemics in the modern age. de Waal tells this story through chronological case histories built around pathogen ‘protagonists’, personified in order to make the particularity of their properties more tangible. Cholera, influenza and HIV/AIDS each receive their own chapters, with yellow fever, typhus and Ebola playing significant supporting roles. Each of these chapters, as well as the one on the pandemic preparedness ahead of COVID-19, are highly valuable as introductions to the social history of medicine. In each case, de Waal captures the intricate web of ecology, pathology, social and political conditions, and cultural experiences that combine into what he calls the ‘pandemy’, that is, the social crisis in its entirety as opposed to simply the global spread of one infectious agent. These chapters answer the great need for accessible, provocative and insightful course literature that helps make sense of our current pandemic experience.

Disease has accompanied military action throughout human history, and military action has frequently been involved in response to epidemic outbreaks and the accompanying civil unrest. However, de Waal argues that the ‘idea that science could declare war on disease, and win’ (p. 62) originated in Germany in the 1880s in connection with Robert Koch’s isolation of the cholera bacillus. Crucially, according to de Waal, this idea was not just a scientific metaphor, but was incorporated into a new public health paradigm. This new public health was fixated on the germ, and established the expectation of its defeat through targeted medical interventions.

The consequence of the war script, according to de Waals analysis, is that it narrows the view on infectious disease in ways that actually promote the evolution of new, potentially devastating pathogens: Improved military medicine enabled war on a much larger scale, which in the First World War provided ideal conditions for the 1918 influenza. Mass-culling of poultry in response to avian flu in the 1990s benefitted large-scale chicken farms at the expense of smaller chicken farmers, which promoted factory farming in which disease is prone to spread. Drawing from the experiences made in relation to HIV/AIDS and Ebola, de Waal instead argues that the bio-social ‘pandemy’ cannot be halted with the war script, but must be managed with bio-social means rooted in the affected communities.

The book is extremely rich in telling examples of the necessity of a more comprehensive approach to epidemic disease, in line with One Health and Anthropocene perspectives. However, as a monograph de Waals account is at times hard to follow as it struggles to hold the many showcases and fascinating glimpses into history together in two simultaneous narratives: one about the consequences of the war script, and one about his proposed alternative, that is, a narrative of pandemics as bio-social processes. de Waal suggests that the coronavirus pandemic could be an ‘emancipatory catastrophe’. It could be, he suggests, an opportunity to recover some of the convictions of the Nineteenth century anti-contagionists—to again recognise disease as co-produced by pathogens, hosts and environment. This, he argues, would provide a basis for handling not just the next pandemic event, but to address wider and interrelated health, environmental, and social crises.

